# Clinicopathological and mutational characteristics of primary double mutant gastrointestinal stromal tumor: a single center study with review of the literature

**DOI:** 10.1186/s12885-023-10684-x

**Published:** 2023-03-08

**Authors:** Jiaqi Yan, Xin He, Chaoyong Shen, Yan Zou, Huijiao Chen, Yuan Tang

**Affiliations:** 1 Department of Pathology, West China Hospital, Sichuan University, Chengdu, Sichuan China; 2 Department of Gastrointestinal Surgery, West China Hospital, Sichuan University, Chengdu, Sichuan China

**Keywords:** Gastrointestinal stromal tumor, Primary double mutation, Clinical pathological and mutational characteristics, Literature review

## Abstract

**Aims:**

Primary double KIT/PDGFRA mutations are very rare in gastrointestinal stromal tumours (GISTs) but have not been comprehensively studied to date. In the present study, we investigated the clinicopathologic and genetic features of eight cases of primary double-mutant GISTs, and we reviewed the literature.

**Methods and results:**

The tumours occurred in six males and two females (age range 57–83 years) and involved the small intestine (n = 4), stomach (n = 2), rectum (n = 1) and retroperitoneum (n = 1). Clinical manifestations were variable, ranging from indolent (no symptoms) to aggressive disease (tumour rupture and haemorrhage). All patients underwent surgical excision, and six of them were treated with imatinib. No one experienced recurrence or other complications during the follow-up time (10 to 61 months). Histologically, all the tumours exhibited mixed cell types, accompanied by variable interstitial changes. KIT mutations were detected in all cases, and the majority of them were present in different exons (n = 5). No PDGFRA exon 12, 14 or 18 mutations were found. All the mutations were validated by next-generation sequencing, and two additional variants with comparatively low allelic fractions were identified in one case. Two of the cases had available allele distribution data, one with an *in cis* compound mutation and the other with an *in trans* compound mutation.

**Conclusion:**

Primary double-mutant GISTs have distinctive clinicopathologic and mutational features. Studies of more cases are necessary for a better understanding of these tumours.

**Supplementary Information:**

The online version contains supplementary material available at 10.1186/s12885-023-10684-x.

## Introduction

Gastrointestinal stromal tumours (GISTs) are the most common mesenchymal neoplasms of the alimentary tract that originate from Cajal cells and are most commonly located in the stomach and small intestine. Approximately 85% of GISTs harbour gain-of-function mutations in KIT or PDGFRA receptor tyrosine kinase (RTK) genes, and this molecular event leads to constitutive, ligand-independent activation that results in the activation of downstream oncogenic signalling pathways. Imatinib mesylate is a tyrosine kinase inhibitor that is widely used as a first-line therapy for GISTs and has greatly improved the survival time in patients and delayed disease progression [2]. However, the long-term use of imatinib may induce secondary KIT or PDGFRA mutations, representing a mechanism of acquired drug resistance in GISTs [3]. Patients who have not been treated with imatinib or other RTK inhibitors will generally harbour only one mutation, which is linked to tumour pathogenesis. This category of GIST is referred to as primary mutant GIST [4]. Very few double mutations have been identified in primary mutant cases. As only sixteen cases of primary double-mutant GISTs have been sporadically reported in the literature, the clinicopathologic and mutational profiles of such tumours are limited [5–11], and the previously reported cases do not show any obvious differences from generally reported GISTs in terms of clinicopathologic features [9]. Only one study analysed the genotype of double-mutant GISTs and found that mutations were located in the same allele but likely represented separate mutational events [4].

To raise awareness among pathologists and clinicians in regards to recognizing this rare malignancy, herein, eight new cases of double-mutant GISTs at one institution are reported, along with a literature review summarizing the clinicopathologic and gene aberration features of these rare tumours.

## Materials and methods

### Patient selection

A total of 1530 GIST cases were collected retrospectively from the Department of Pathology, West China Hospital of Sichuan University, from June 2011 to December 2020. Haematoxylin and eosin (H&E)-stained slides were reviewed by two pathologists specializing in soft tissue pathology (X.H. and H.-j.C.), and the diagnosis of GIST was based on the previously established morphology and immunohistochemical criteria [1]. All specimens were routinely processed with PCR amplification and Sanger sequencing for KIT and PDGFRA gene analysis. Available clinical details and follow-up data from electronic medical records or patient files were investigated. Patients with tumour metastasis/recurrence or a history of RTK inhibitor treatment were excluded. Thus, eight cases (8/1530, 0.52%) were included in the current study. All patients provided written informed consent for the collection and publication of their medical information during their initial visit to the hospital. This study protocol was approved by the Ethics Committee of West China Hospital of Sichuan University.

### Histopathology and immunophenotyping

H&E-stained sections were used to assess the histopathologic features. The risk category was evaluated on the basis of the NIH 2008 classification system [12]. A panel of antibodies were used as follows: CD117 (YR145, MaiXin), CD34 (EP88, ZhongShan), DOG-1 (SP31, MaiXin), SMA (UMAB237, ZhongShan), Desmin (MX046, MaiXin), S-100 (4C4.9, MaiXin), and Ki-67 (MIB-1, DAKO). The staining was processed in accordance with the manufacturer’s protocols. Positive and negative controls were employed. Sections were imaged using brightfield on the KF-PRO-020 slide scanner (KONFOONG biotech international CO., LTD).

### Molecular analysis

DNA from paraffin-embedded tissue samples was extracted using the QIAamp DNA FFPE Tissue Kit (Qiagen Inc., Valencia, CA, USA) following the manufacturer’s instructions. Tissue sections were selected by a pathologist (H.-j.C.) prior to DNA isolation to confirm that the percentage of tumour cells was always greater than 20%. The DNA concentration was measured with a Qubit dsDNA assay (Life Technologies, CA, USA).

For Sanger sequencing, PCR conditions and direct sequencing of KIT exons 9, 11, 13, and 17 and PDGFRA exons 12, 14, and 18 were performed using methods as previously described [6, 11]. The PCR products were purified using the QIAquick PCR purification kit (Qiagen Inc.) and were sequenced in both directions using the Applied Biosystems 3730XL DNA analyser (Applied Biosystems, Foster, CA) according to the manufacturer’s instructions. The purified samples were visualized using Chromas2 (Technelysium Pty. Ltd, Helensvale, Australia), and the sequences were analysed following alignment with NCBI Reference Sequences for KIT and PDGFRA genes.

Next-generation sequencing was used for analysis of cis/trans status of KIT/PDGFRA mutations. For its assay, DNA was sheared using Covaris M220. The sheared tissue DNA underwent end repair, phosphorylation and adaptor ligation. Fragments of 200–400 base pairs (bp) were selected using Agencourt AMPure beads (Beckman Coulter, Brea, CA, USA) followed by hybridization with capture probe baits, hybrid selection with magnetic beads and PCR amplification. A bioanalyzer high-sensitivity DNA assay was performed to assess the quality and size of the fragments. A minimum of 50 ng of DNA was used for library construction. Twelve PCR cycles were used for library amplification. The indexed samples were sequenced on Nextseq500 (Illumina, Inc., San Diego, CA, USA) with paired-end reads with read length of 150 bp.

The genomic profiles were assessed by performing capture-based targeted deep sequencing using the ColonCore panel (Burning Rock Biotech, Ltd.), which included the KIT and PDGFRA genes (Supplemental Table 1). Sequencing data were mapped to the human genome (hg19) using BWA 0.7.10. The alignment was further refined by the functions of local realignment, base quality recalibration and indel realignment provided by GATK 3.2. The variants were called on the final alignment file (BAM) with VarSCan. All SNVs and indels were annotated with ANNOVAR and were manually confirmed by visual inspection with the Integrative Genomics Viewer (IGV) tool.

### Mutations interpretation

Identified KIT/PDGFRA mutations were categorized according to the oncogenicity SOP(standard operating procedure) from the Clinical Genome Resource(ClinGen), Cancer Genomics Consortium(CGC) and Variant Interpretation for Cancer Consortium(VICC) [12]. We collected the evidences for interpretation with databases including NCBI ClinVar database, LOVD database and COSMIC. Variants can be classified as oncogenic, likely oncogenic, uncertain significance(VUS), likely benign or benign.

## Results

### Clinical manifestations

**Fig. 1:**
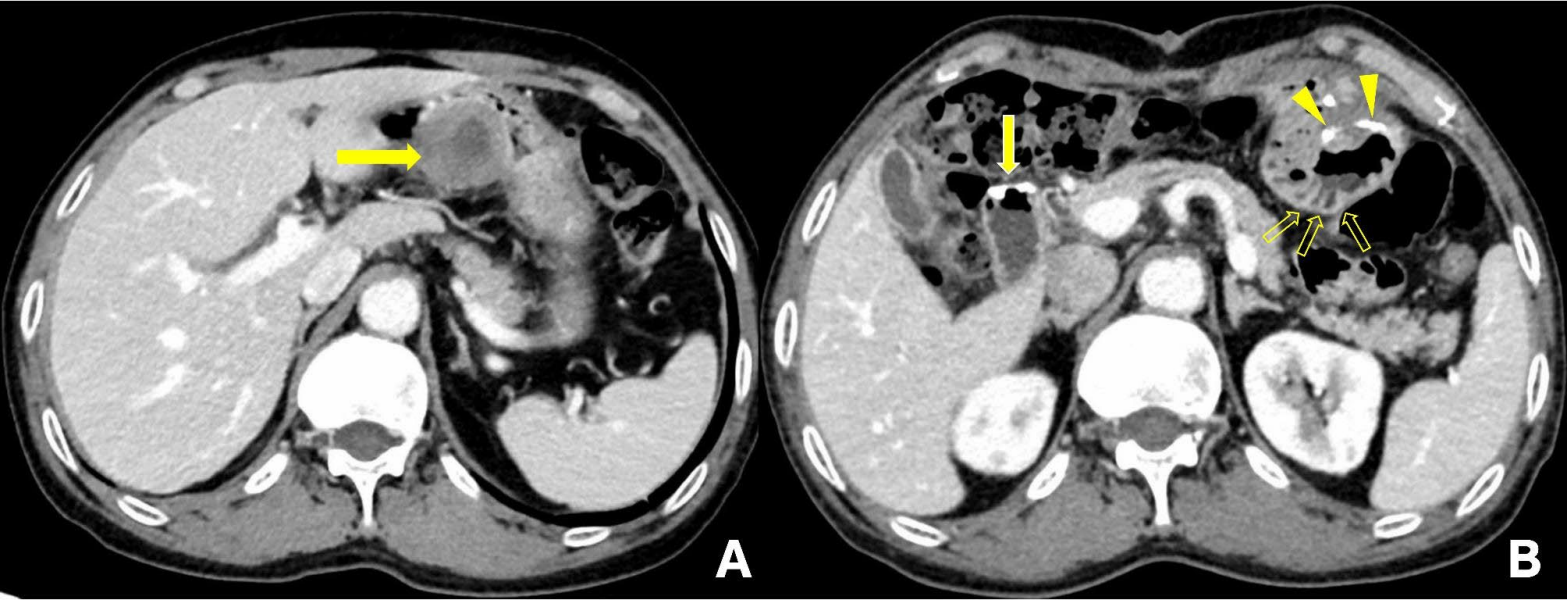
Contrast-enhanced abdominal computed tomography images(case 6). A: Preoperative CT imaging (December 2019): CT scan shows a large, exophytic mass with heterogeneous enhancement in the wall of the distal stomach. B: Postoperative CT imaging following Billroth II gastrojejunostomy (September 2021): CT scan shows surgical staples at the gastrojejunal anastomosis site(arrowheads), where no local tumor recurrence was observed. The jejunal loop(open arrows) characterized by the valvulae conniventes is also noted. Metallic suture material(solid arrow) clearly indicates the duodenal stump

The clinical characteristics of the eight patients with primary double-mutant GISTs are listed in Table 1. The patient age varied from 57 to 83 years, with a median age of 63.5 years. The male to female ratio was 3:1 (six males and two females). The clinical presentation varied: 3 patients were asymptomatic, and tumours were detected by screening examination with a gastrointestinal endoscope; 2 patients had a palpable abdominal mass, and in particular, one of them was admitted to the hospital for tumour rupture causing acute diffuse peritonitis; 2 patients presented with abdominal pain; and 1 patient presented with haematochezia and a change in bowel habits. Tumours involved the small intestine (4 patients), stomach (2 patients), rectum (1 patient) and retroperitoneum (1 patient). According to the 8th edition of AJCC staging system, 3 patients were in stage II, 2 were in stage IB, 2 were in stage IIIB and 1 was in stage IIIa. All the patients underwent surgical excision as the primary treatment, and the specimens were submitted for pathologic examination and molecular study. Two patients did not receive RTK inhibitor therapy any time after surgery, and the other 6 patients were treated with adjuvant imatinib therapy(200-600 mg). The median duration of imatinib was 28 months(range, 8-40months) and adverse effects included edema(patient 4 and 5), mild anemia and thrombocytopenia(patient 8). Complete clinical follow-up data were available for all patients. The median follow-up time was 28.5 months, ranging from 10 to 61 months. Abdominal enhanced CT re-examination was conducted regularly (cases 1–3 and 5: every 6 months; other cases: every 3 months). No patient was found to have any evidence of recurrence or metastasis (Fig. 1) of the disease during the follow-up period, and all of them were alive at the last follow-up (October 2021).

**Table 1:** Clinicopathologic and histologic features of double primary mutant GISTs

Case	Age	Gender	Clinical Presentation	Site	Size (Maximum Diameter)	Mitosis (50/HPF)	NIH Risk Classification	AJCC stage	Cell type (histologic subtype)	Treatment	Follow-up Information
1	63	M	hematochezia	rectum	6 cm	5	High	II	Mixed(hypercellular spindle + hypercellular epithelioid)	excision and imatinib(400 mg)	NED at 61 mo
2	69	M	asymptomatic	ileum	5.5 cm	4	High	II	Mixed(sclerosing spindle + sclerosing epithelioid )	excision and imatinib(400 mg)	NED at 45 mo
3	63	F	asymptomatic	stomach	5.2 cm	2	Moderate	IB	Mixed(sclerosing spindle + sclerosing and dyscohesive epithelioid)	excision and imatinib(200 mg)	NED at 43 mo
4	83	M	abdominal mass	jejunum	21.3 cm	10	High	IIIB	Mixed(hypercellular spindle + hypercellular epithelioid )	excision and imatinib(400 mg)	NED at 39 mo
5	62	M	abdominal pain	duodenum	7 cm	8	High	IIIB	Mixed(sclerosing spindle + dyscohesive and sclerosing epithelioid)	excision and imatinib(400 mg)	NED at 22 mo
6	70	M	asymptomatic	stomach	6 cm	2	Moderate	IB	Mixed(sclerosing spindle + dyscohesive epithelioid)	excision	NED at 21 mo
7	57	F	abdominal pain	jejunum	5.4 cm	2	High	II	Mixed(sclerosing spindle + sclerosing and dyscohesive epithelioid)	excision	NED at 12 mo
8	64	M	abdominal mass	retroperitoneum	10.5 cm	2	High	IIIA	Mixed(hypercellular and palisade-vacuolated spindle + epithelioid)	excision and imatinib(600 mg)	NED at 10 mo

Abbreviations: HPF, high power field; M, male; F, female; NED, no evidence of disease;

### Morphology

**Fig. 2:**
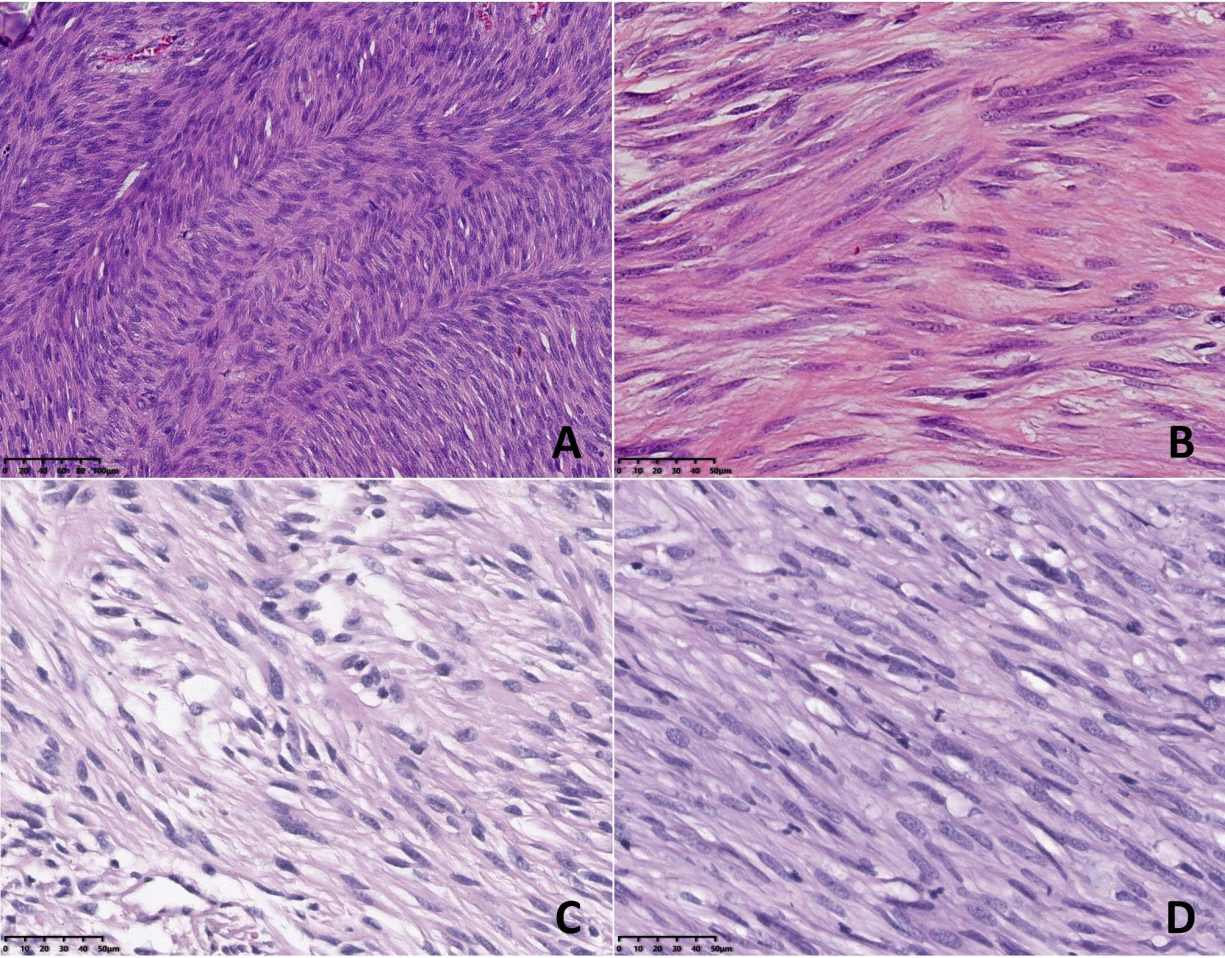
Histological features of primary double-mutant GISTs. A-D: Cell morphology of the spindle cell components of the mixed cell type. A: Hypercellular spindle cell pattern: the spindle cell tumour was composed of uniform and densely packed spindle cells with cigar-shaped nuclei, showing a fascicular and interlaced arrangement (case 1). B-D: Cell morphology of the spindle cell components of the mixed cell type. B, C: Sclerosing spindle cell pattern: slender spindle cells dispersed in a sclerosing stroma (case 6, case 7). D: Palisading-vacuolized cell pattern (case 8). [Original magnification 200× in panel (A), 400× in panels (B-D)]

**Fig. 3:**
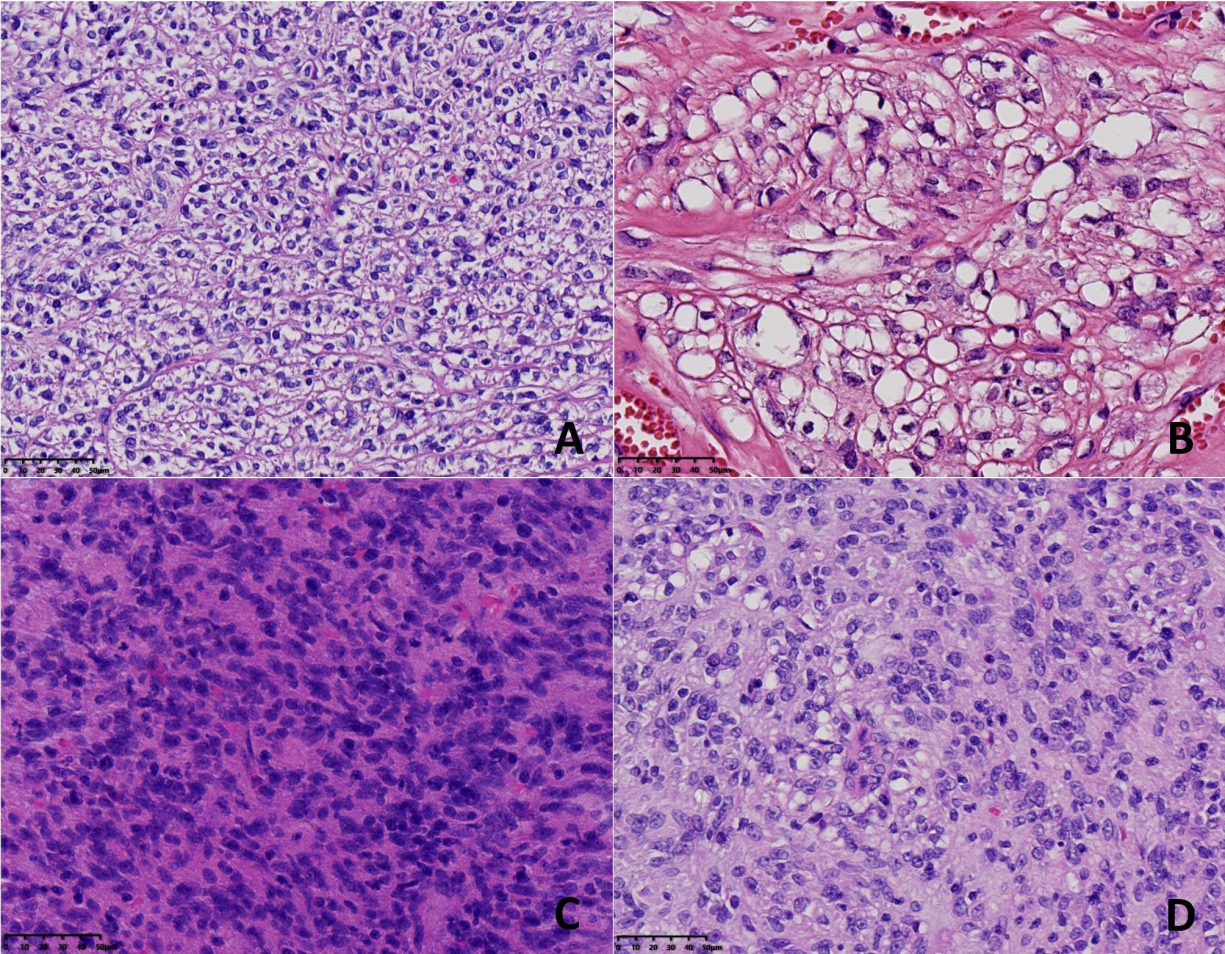
Histological features of primary double-mutant GISTs. A-D: Cell morphology of the epithelioid cell components of the mixed cell type. A: Discohesive epithelioid pattern: round-to-polygonal cells with abundant clear cytoplasm showing distinct cell membranes (case 5). B: The epithelioid cells contain prominent cytoplasmic vacuoles, which impart a signet ring appearance (case 3). C: Hypercellular epithelioid pattern: diffuse sheets of small epithelioid cells with less distinctive cellular borders (case 4). D: Sclerosing epithelioid pattern: round-to-oval cells set in a syncytial pattern with a sclerosing stroma (case 7). [Original magnification 400× in panels (A-D)]

**Fig. 4:**
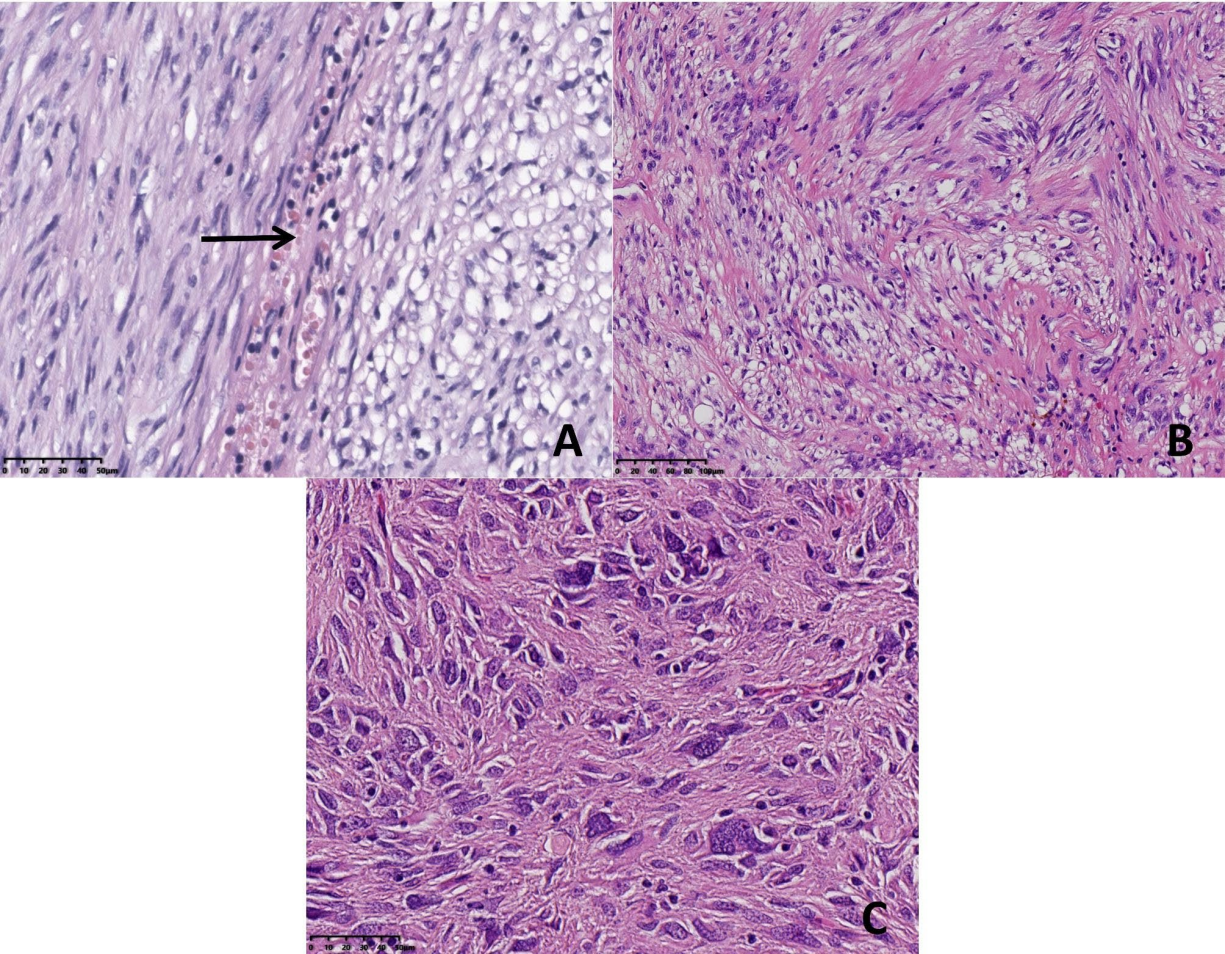
A: The spindle cell area and epithelioid cell area exhibited a transition border (case 8). B, C: Intermingled with each other (case 4, case 2). C: The short spindle cells and epithelioid cells mixed with each other in a collagenized background without distinctive cell borders. Tumour cells showed moderate nuclear atypia, and mononucleate and multinucleate tumour giant cells were noted (case 2). [Original magnification 200× in panels (B), 400× in panels (A, C)]

**Fig. 5:**
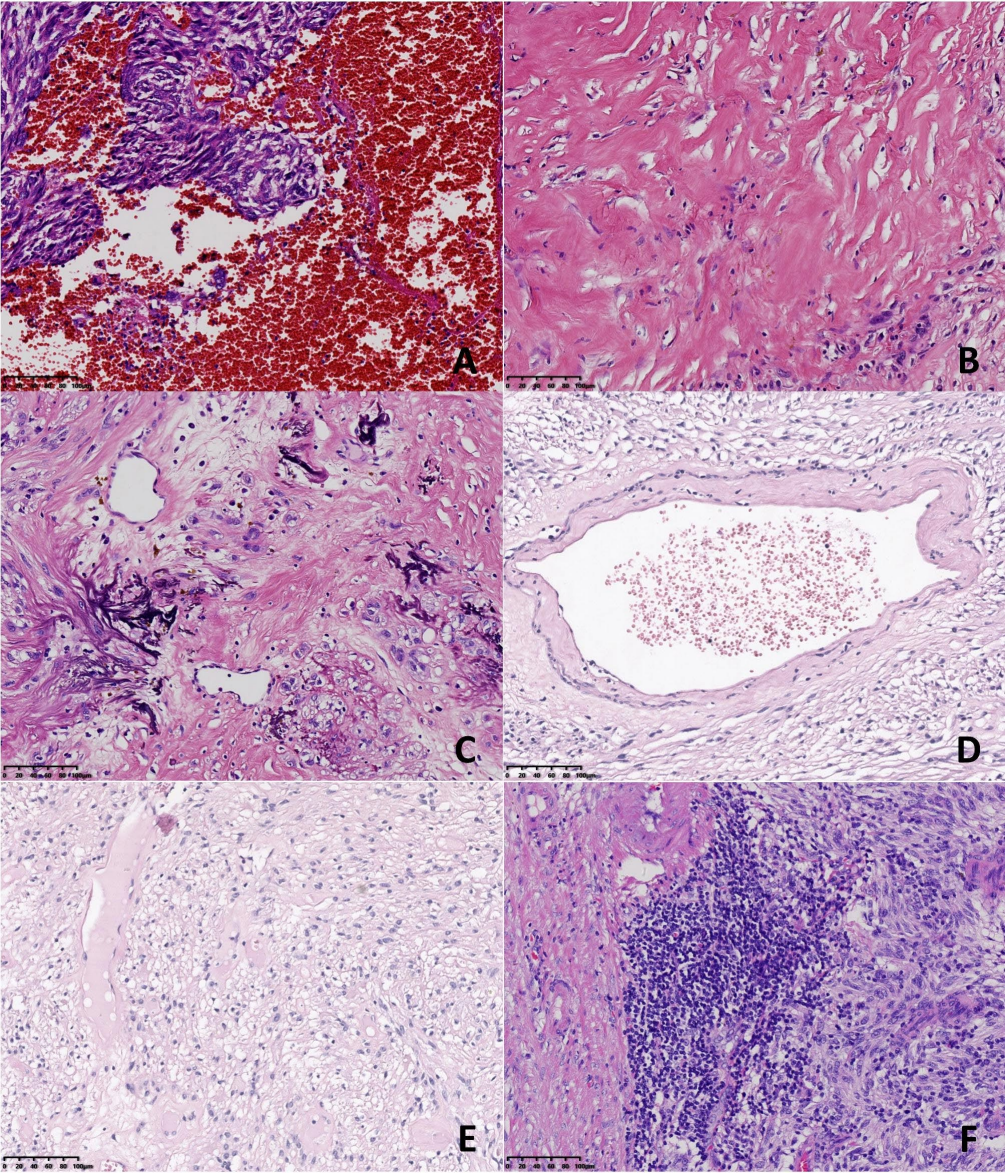
Other morphologic variations. (A) Intratumoral haemorrhage (case 4). (B) Extensive collagen deposition (case 3). (C) Scattered foci of calcification (case 6). (D) Hyalinized and dilated blood vessels (case 7). (E) Myxoid degeneration (case 7). (F) Focal lymphocytic aggregation (case 5). [Original magnification 200× in panels (A-F).]

The morphologic features of the neoplasms in the 8 cases are summarized in Table 2. Grossly, the resected tumours presented as circumscribed or nodular solid masses. The cut surface was soft and grey-white in colour, with foci of haemorrhage and necrosis. Tumour rupture was found in case 4. The size ranged from 5.2 to 21.3 cm, with an average of 8.3 cm. Histopathologically, all 8 cases were of the mixed cell type. Both spindle cells and epithelioid cells presented several histologic patterns (Table 1). Among the spindle cell components, there were the hypercellular subtype, sclerosing subtype and palisade-vacuolated subtype (Fig. 2). Epithelioid cell morphology was subclassified as discohesive, sclerosing and hypercellularity (Fig. 3). Cytoplasmic vacuolization was commonly seen in epithelioid cells, resulting in fat-containing or signet-ring cell formation. In 4 cases of mixed cell type, one of each cell component contained an admixture of 2 patterns that varied from one microscopic field to another. The different spindle and epithelioid cell tumours were intermingled with each other or exhibited a transition border (Fig. 4). Nuclear atypia was mild in 5 cases and moderate in 3 cases. Despite the basic histologic patterns described above, other accompanying morphological findings were collagen deposition, focal haemorrhage, focal lymphocyte aggregation, calcification, myxoid degeneration and vascular changes, such as dilated vessels and perivascular hyalinization (Fig. 5). In part, tumour cells were distributed focally in a densely sclerotic and hyalinized collagenous stroma. Skenoid fibres were not observed in any of the cases. Mitotic counts varied from 2 to 10 per 50 HPFs (median, 4/50 HPFs). According to the risk categories stratified based on the NIH 2008 system, 6 cases were classified as high risk, and 2 cases were classified as moderate risk.

**Table 2:** Immunohistochemical and genetic findings of double primary mutant GISTs

	Immunohistochemistry						Ki−67	KIT/PDGFRA mutation analysis	
Case	CD117	CD34	DOG−1	SMA	Desmin	S−100		Mutations	Allelic distribution
1	+	+	+	-	-	-	5–8%	KIT exon 11 p.V559D + exon13 p.K642E	NA
2	+	-	+	+, scattered	-	-	10%	KIT exon 11 W557R + D579del	trans
3	+	+	+	-	-	+, scattered	1%	KIT exon 11 V569_L576del + exon 13 M651L KIT exon 3 L138fs* + exon 13 K642fs*	NA
4	+	ND	-	+, focal	-	-	10%	KIT exon 11 V559A + exon 13 I653fs	NA
5	+	+	+	-	-	-	10–15%	KIT exon 11 V560G + D579A	cis
6	+	+	+	+, scattered	-	-	5–10%	KIT exon 11 V559D + V559G	NA
7	+	+, focal	+	+, focal	-	-	5%	KIT exon 13 K642E + exon 17 S821C	NA
8	+	+	+	-	-	-	5–10%	KIT exon 9 A502_Y503dup + exon 11 W557_V560delinsC	NA

NA, not available;

*The mutations were only present in a proportion of the DNA molecules identified by NGS (AF < 10%).

### Immunophenotypes and mutation analysis

IHC staining data and details of the molecular findings are summarized in Table 2. The tumour cells of all cases were positive for CD117 and DOG-1, while CD34 was absent in one case (case 2). SMA was present in three cases, and desmin was negative in all cases. S-100 was present in one case (case 3). The Ki-67 index ranged from 1 to 15%.

In the eight cases with KIT mutations, both mutations were present in exon 11 in three cases (cases 2, 5 and 6); the mutations were present in exons 11 and 13 in two cases (cases 1 and 3), in exons 13 and 17 in one case (case 7) and in exons 9 and 11 in one case (case 8). None of these cases presented PDGRFA mutation. Regarding mutation subtype, the predominant KIT exon 11 mutant was single point mutations that involved three codons, 559, 560, and 579, and the other genotype was deletions that affected codons 557–560, 569–576 and 579. Mutations located in exon 13 affected codons 642, 651 and 653. The mutation in exon 9 was an AY502-503 duplication. One mutation has not yet been described in GISTs: a 2 bp (TT) deletion at codon 653 in exon 13 (I653fs).

**Fig. 6:**
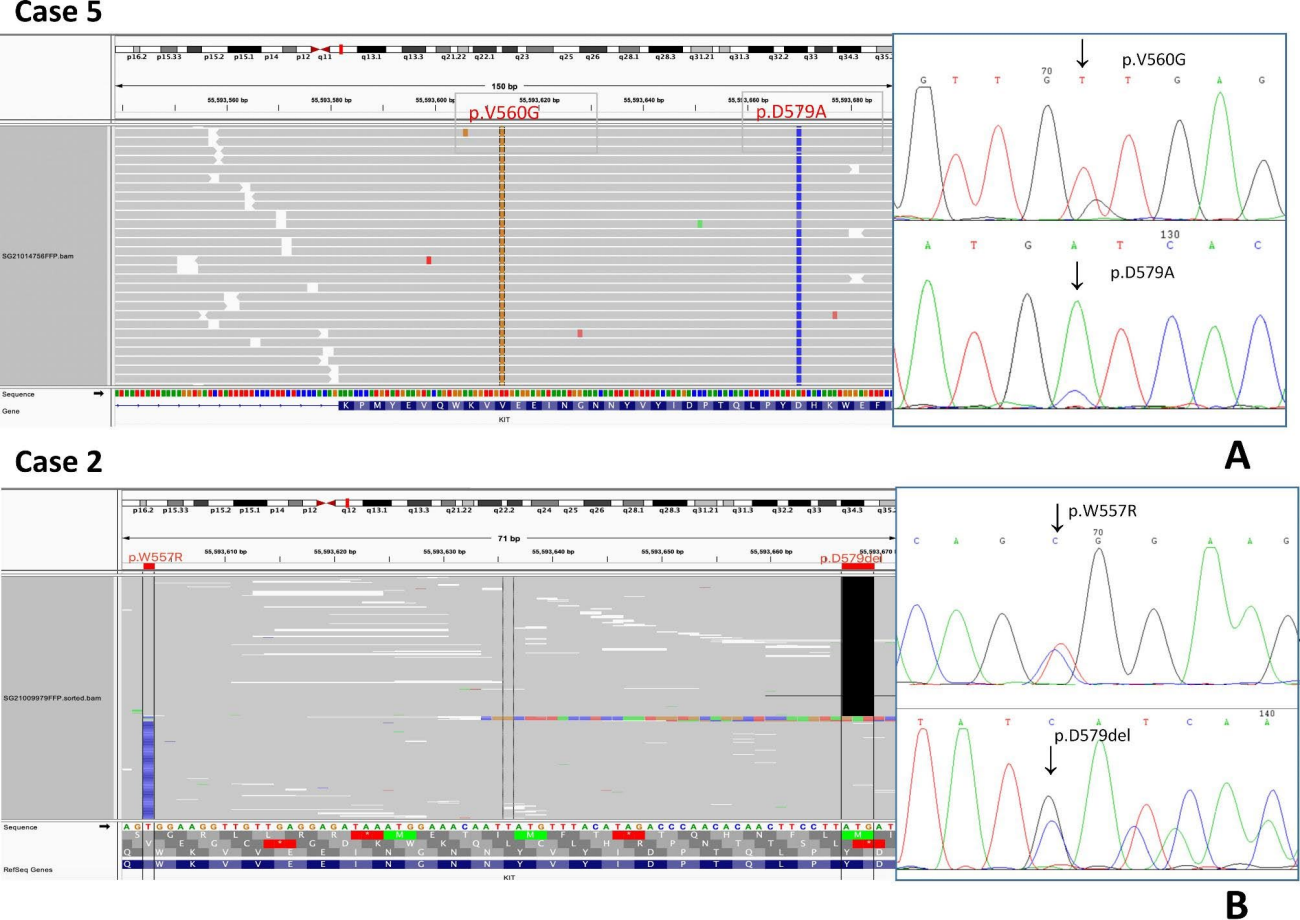
Left: IGV tool output of NGS: (A) Mutations *in cis* on the same allele (case 5). (B) Mutations *in trans* on different alleles (case 2). Right: Sanger sequencing chromatogram of mutational analysis: (A) Mutations in exon 11 (V560G and D579A) (case 5). (B) Mutations in exon 11 (W557R and D579del) (case 2)

For Sanger sequencing, both alleles are sequenced in one reaction together, and it is difficult to determine if the 2 variants detected are in the same allele (cis) or in different alleles (trans). Thus, we screened 7 of the cases (case 8 did not have eligible paraffin-embedded samples) for cis/trans polymorphism of KIT status using capture-based targeted sequencing. Two cases had an evaluable configuration: one harboured KIT mutations in different alleles, and the other harboured mutations in cis (Fig. 6). In case 6, mutations were in the same sites (V559D and v559G), making it more likely that the two mutations were distributed on different reads. Three cases could not be evaluated because the length between the two mutations was longer than 300 bp. In addition, NGS revealed 2 additional variants of KIT in case 3, which were L138fs (allelic frequency, AF: 5.57%) and K642fs (AF: 5.76%) in case 3 and K642E(AF: 30.82%) in case5 (Supplementary Table 2).

In addition, to identify whether double mutations associated with the different cell component of the tumour, we performed micro-dissection to discrete subpopulations of cells and found that they exhibit same mutations on sequencing(Supplementary Fig. 1). These results suggest that cases of double-mutant GIST are unlikely due to anatomically overlapping tumours bearing distinct single oncogenic mutations, and further that regions of tumour exhibiting different morphologies contain the same double mutation.

### Clinical classification of variants

We analyzed the oncogenicity of all these double variants and found that three cases(case 1, case 2 and case 6) have two distinct oncogenic KIT mutations, whereas the other five cases do not: case 3(exon 11 V569_L576del, oncogenic; exon 13 M651L, VUS); case 4(exon 11 V559A, oncogenic; exon 13 I653fs, VUS); case 5(exon 11 V560G, oncogenic; D579A, VUS); case 7(exon 13 K642E, oncogenic; exon 17 S821C, VUS); case 8(exon 9 A502_Y503dup, oncogenic; exon 11 W557_V560delinsC, VUS).

## Discussion

As most cases from the literature lack detailed clinical pathological data, we describe a series of clinical, morphological and immunohistochemical characteristics of primary double-mutant GISTs from a single institution. Concomitant KIT/PDGFRA mutations are often encountered after RTK inhibitor therapy, whereas a therapy-naive GIST with a double mutation is rare, and the reported incidence varies. Rossi et al. studied the mutation status of 135 micro-GISTs (< 2 cm) without imatinib treatment and found that 3 cases (2.2%) harboured a double KIT mutation [6]. A large cohort involving 1177 GIST biopsies across 4 UK centres revealed that 9 cases (0.7%) had a primary double mutation [9]. According to other mutational investigations of primary GISTs, double-mutant GISTs account for only 0.42–2.2% [5–[6, 8, 10]–11]. In the present cohort, the frequency is in the lower range of previously reported series, only 0.52% (8/1530) of the cases were putative primary double-mutant GISTs and 0.196%(3/1530) were with two established oncogenic mutations. Such a relatively low rate of double mutation may be because our mutational analysis was routinely performed with Sanger sequencing. Although this technique remains the main validation method for detecting variants, its limitations may lead to the absence of some low-abundance mutations. By NGS analysis, one case in our series was identified as having an additional variant in exon 13, namely, p.K642fs (AF: 5.76%), which Sanger sequencing failed to detect. Wong et al. validated double-mutant cases by NGS and identified additional variants in 2 cases [9]. Moreover, we also identified a case with another mutation in exon 3 that was not evaluated by routine Sanger sequencing. Wong et al. pointed out that a second mutation may have been missed because some centres only test the more commonly mutated exons first, and the remaining exons would not be tested unless these were wild type [9]. Another additional variant, K642E in case 5, was detected at a high frequency using NGS but failed to detected by sanger sequencing. It is considered that tumor cell mutational heterogeneity may caused the inconsistency.

As shown in Table 3, there has not been sufficient information about the clinicopathologic data of reported primary double-mutant GISTs. From the limited available data, this malignancy primarily affected middle-aged men (median age, 59.5 years; sex ratio, 2.67:1), and the stomach (10/13, 76.9%) was the most likely site of occurrence, while the small intestine (2/13, 15.4%) and other sites (1/13, 7.7%) were uncommon. The size varied from micro-GISTs (0.8 cm) to tumours of 35 cm, with the mean size being 10 cm, and the mitotic index was variable, as low as 1/5 mm^2^ but ranging up to 60/HPF. The tumours were categorized into moderate-risk (2/5, 40%) and high-risk groups (3/5, 60%). Seven patients were followed-up, among whom 2 had received imatinib therapy. Two patients died, and details were not available for one patient, whereas the survival time of the other was 7 years, and this patient’s death was unrelated to GIST. Metastasis was noted in one case after 3 years. Three patients were alive for at least 23, 36 and 64 months, respectively. The current series displayed similarities in age and sex but differences in some aspects. Most tumours tended to arise in the bowel (6/8, 75%), including the small intestine, duodenum and rectum. Most tumours (6/8, 75%) were of high risk, which was slightly higher than the reported primary double-mutant cases. Large tumour size and high mitotic rate are generally associated with poor prognosis, and high-risk patients have more than 15–20% risk of disease recurrence [13, 14]. However, the patients in our cohort showed a favourable outcome, and all of them were alive and free of disease during the follow-up period.

**Table 3:** Clinicopathological features in the previously reported 16 cases in literature

Author	No.	Gender	Age	Tumor site	Size	Morphologic features	Risk class	Mutation	Outcome
Heinrich [5]	1	M	63	stomach	NS	NA	NA	KIT exon 13 K642E + exon 17 N822H	DOD
Rossi [6]	2	NA	NA	stomach	0.8 cm	NA	NA	KIT exon 11 P551-M552delinsL + W557G	NA
	3	NA	NA	small intestine	1.7 cm	NA	NA	KIT exon 9 K509I + G510del	NA
	4	NA	NA	stomach	2 cm	NA	NA	KIT exon 9 N505H + K509I	NA
Conca [7]	5	M	68	stomach	> 5 cm	mixed	High(NIH2002)	KIT exon 11 W557G + Y578C	NED, 36 m
Kalfusova [8]	6	NA	NA	NA	NA	NA	NA	PDGFRA exon 14 M642T + N659H	NA
Wong [9]	7	M	45	stomach	3.5 cm	spindle	Moderate(AFIP)	KIT exon 11 W557-I563del + T574-Q575delinsK	Alive(NA for details), 23 m
	8	F	56	small intestine	19 cm	mixed	High(AFIP)	KIT exon 11 W557C + V560del	Metastasis after 3 years
	9	M	49	stomach	NS	spindle	NA	KIT exon 11 Y568D + Y578C	Alive(NA for details), 64 m
	10	F	71	NA	NA	spindle	NA	KIT exon 11 Y568D + L576P	NA
	111	M	50	stomach	NA	mixed	NA	PDGFRA exon 18 D842V + M844L	NA
	12	M	70	stomach	35 cm	spindle	Moderate(AFIP)	KIT exon 17 N822K + K826Q	DOD, 7 years
	13	F	43	pelvis	NA	spindle	NA	KIT exon 11 K558N + V559A	NA
	14	M	63	stomach	13 cm	spindle	High(AFIP)	KIT exon 11 W557G + K558E	NA
Haefliger [10]	15	M	NA	stomach	NA	spindle	NA	KIT exon 11 V559A + V560D	NA
Bombac [11]	16	NA	NA	NA	NA	NA	NA	PDGFRA exon 14 K627E + N659H	NA

Abbreviations: NA, not available; M, male; F, female; DOD, died of disease; NED, no evidence of disease;

In the histopathologic findings, we found that histologic subtyping of tumours indicated that all of the tumours were mixed-cell tumours. These observations were different from those of reported primary mutant GISTs and general GIST populations, in which the majority were spindle type [15, 16]. In the WHO classification of tumours of soft tissue and bone (2020), most GISTs are spindle cell type, with epithelioid morphology accounting for approximately 20–25%, and only a small number of cases feature a mixed-cell type [17]. However, Jumniensuk et al., Li et al., Cao et al. and Tazawa et al. found that the mixed-cell type was more common in Asian populations [18–21], indicating that the histologic cell type may have some connection with ethnicity. The basic histologic patterns (sclerosing, discohesive and hypercellular) of the mixed-cell type in our series were more varied than those of mixed-cell GISTs previously described by Jumniensuk et al. and Lopes et al [18, 22].

All the reported cases harboured primary double mutations in the same exon of the same gene except one described mutation in KIT exon 13 and exon 17. The most frequent mutations were those in KIT exon 11 (9/16, 56.3%), followed by 2 with KIT exon 9 mutations, 1 with PDGFRA exon 14 mutations, 1 with PDGFRA exon 18 mutations and 1 with KIT exon 17 mutations. In contrast to the literature, most of our cases carried double mutations involving different exons of the KIT gene (5/8, 62.5%). The majority of them were localized in exon 11 between codons 550 and 580, which is the commonly mutated region and similar to that genotype, which is generally reported in GISTs [23, 24]. Notably, we identified a novel mutation in exon 13, p.I653fs(c.1958_1959delTT) which resulted in a frameshift mutation and may lead to a protein length change in the proximal kinase domain (ATP-binding section). Most of the deletions, insertions and duplications in KIT exons invariably remain in frame. Only two GIST cases with a KIT exon 13 frameshift mutation has been reported in the literature [25, 26]. Both of them were p.K642fs mutations resulting in sequence termination and a truncated KIT protein lacking the C-terminal component. Furthermore, one of these studies found this mutation to confer imatinib resistance, which was identified by cell culture experiments [26].

Mutation status also predicts the response to imatinib, and approximately 40–50% of tumours develop secondary drug resistance within 2 years of the start of therapy [27]. Six of our patients received imatinib therapy, of which the longest disease-free survival period was 5 years, indicating that they experienced clinical benefit from the treatment. Wong et al. reported that only one patient in their series received imatinib, and the patient’s recurrent GIST also showed clinical sensitivity to the drug [9]. Conca et al. reported a primary GIST with double mutations in exon 11 (W557G and Y578C) that responded well to imatinib therapy. In silico and in vitro experiments revealed that the double mutation displayed constitutive phosphorylation of the KIT receptor, which was similar to the effect of each single mutation and therefore suggested that mutations on the same molecule do not alter each individual mutation’s effect on KIT receptor activation and sensitivity to imatinib [7]. However, the imatinib susceptibility of both mutations was also found to be different from either of the two mutations. Heinrich et al. reported that a primary double-mutant case (K642E and N822K) experienced an objective partial response, and they tested the in vitro sensitivity of mutant kinases to imatinib and found that coexpression of the imatinib-sensitive K642E mutation with N822K resulted in moderate imatinib resistance, which differed from the isolated K642E isoform [5]. Similarly, with regard to the case of McDonnell et al., the two mutations (V559A and N822I) in melanoma that are individually sensitive to imatinib may demonstrate different responsiveness when they occur in tandem [28]. Although the general mechanism of double mutation that varies with imatinib therapy is still uncertain, we speculate that the two mutations within different functional domains may affect the active conformation of the kinase domain and binding of imatinib to the receptor. In addition, we examined the allelic distribution of the two variants. Previous reports of allelic analysis of primary double-mutant GISTs revealed that the mutations all occurred in the same allele. Although *in cis* mutations within an exon were considered a single complex mutational event, Wong et al. studied a series of GISTs with multiple variants and found that these variants in the same exon or allele possibly represent separate mutational events [9]. In our study, one case had mutations on a different allele, which likely indicates that the double mutations were separate events and that the tumour was functionally homozygous due to loss of the wild-type allele. Lasota et al. found that homozygous KIT exon 11 mutations were associated with a malignant course of disease and supported the hypothesis that the presence of the KIT-WT allele can moderate the effect of a KIT-MT allele [29]. In contrast, in our series, all patients were alive with no evidence of disease at their last follow-up. No significant difference in treatment responses was observed between patients with *in cis* and *in trans* mutations, which is in accordance with the perspective that the sensitivity of trans-positioned mutations to imatinib may be similar to that of corresponding cis-positioned mutations reported by Nishida et al [30]. Therefore, whether double mutations affect KIT receptor activation and sensitivity to imatinib is still unclear.

In terms of the classification of variants, there are some limitations in this study. Because several cases do not bearing two established oncogenic mutations(case 3–5, case 7 and 8), which makes the double mutations in these cases can be defined as “putative double mutations”. Though the eight cases were initially considered to be divided into two subgroups, the cohort size were too small to do further analysis and the phenotype(pathological features, therapeutic response and prognosis) were similar in each case. Also, the second mutations of the “putative double mutations” were all VUS, which needs reclassification and verification. So in this study we did not describe the cases separately and focus on the clinicopathologic characteristics. Further evaluation regarding computational prediction algorithms, protein-structure analysis and functional experiments would ascertain the oncogenicity of the double mutations.

## Conclusion

In conclusion, we present a case series of eight patients with double-primary tyrosine kinase mutations at a single centre. In our cohort, the clinicopathologic characteristics were different from those of reported primary double-mutant GISTs. They predominantly affected the bowel, showed mixed cell morphology and were included in the high-risk category. The double mutations that exist in different KIT exons are unique. The lack of relapse or metastasis indicates that they may have favourable biologic behaviour and sensitivity to imatinib, and further studies are needed to ascertain the relationship between double mutations and the response to TKIs.

## Electronic supplementary material

Below is the link to the electronic supplementary material.

Supplementary Material 1

Supplementary Material 2

Supplementary Material 3

## Data Availability

All data generated and analyzed during the current study are available upon reasonable request. Please contact Prof. Yuan Tang(1202ty@163.com).

## Abbreviations

GIST: Gastrointestinal stromal tumor
RTK: Receptor tyrosine kinase
NGS: Next-generation sequencing
TKI: Tyrosine kinase inhibitor
IGV: Integrative Genomics Viewer
