# Supplementary figures and images for: Clinicopathological and mutational characteristics of primary double mutant gastrointestinal stromal tumor: a single center study with review of the literature
Source: BMC Cancer. 2023 Mar 8;23:217. doi: 10.1186/s12885-023-10684-x (PMC9993699; doi:10.1186/s12885-023-10684-x)

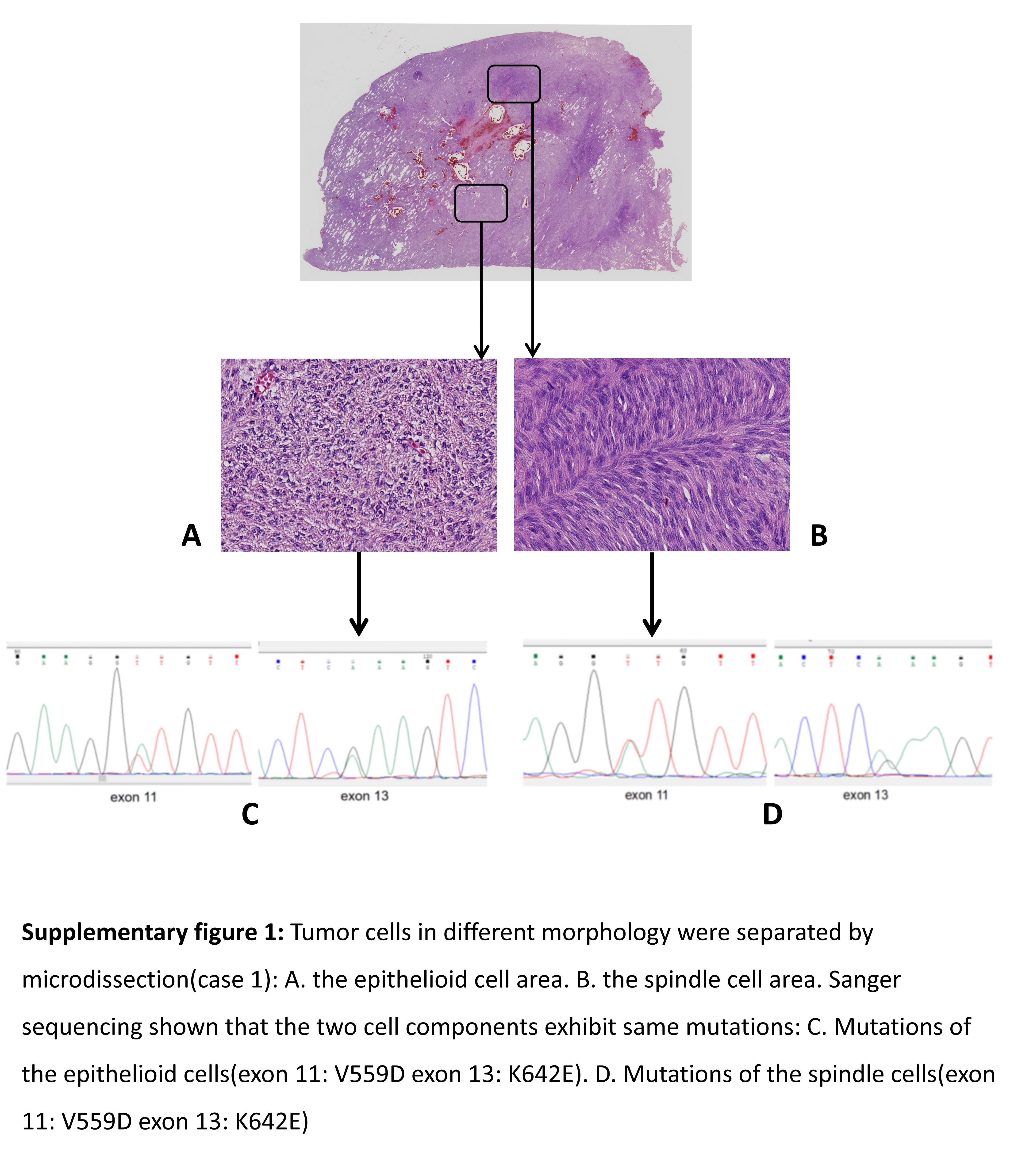

Supplement: Supplementary file 3 — Supplementary Material 3 [file 12885_2023_10684_MOESM3_ESM.jpg]
